# *Trypanosoma cruzi* Mexican Strains Differentially Modulate Surface Markers and Cytokine Production in Bone Marrow-Derived Dendritic Cells from C57BL/6 and BALB/c Mice

**DOI:** 10.1155/2019/7214798

**Published:** 2019-09-15

**Authors:** Cecilia Gomes Barbosa, Tamires Marielem Carvalho Costa, Chamberttan Souza Desidério, Paula Tatiana Mutão Ferreira, Mariana de Oliveira Silva, César Gómez Hernández, Malú Mateus Santos, Rafael Obata Trevisan, Weslley Guimarães Bovi, Virmondes Rodrigues, Juliana Reis Machado, Luiz Eduardo Ramirez, Carlo José Freire de Oliveira, Marcos Vinícius da Silva

**Affiliations:** ^1^Laboratory of Parasitology, Department of Immunology, Microbiology and Parasitology, Federal University of Triângulo Mineiro, Uberaba, MG, Brazil; ^2^Laboratory of Immunology, Department of Immunology, Microbiology and Parasitology, Federal University of Triângulo Mineiro, Uberaba, MG, Brazil; ^3^Department of General Pathology, Federal University of Triângulo Mineiro, Uberaba, MG, Brazil

## Abstract

Dendritic cells (DCs) are a type of antigen-presenting cells that play an important role in the immune response against *Trypanosoma cruzi*, the causative agent of Chagas disease. *In vitro* and *in vivo* studies have shown that the modulation of these cells by this parasite can directly affect the innate and acquired immune response of the host in order to facilitate its biological cycle and the spreading of the species. Many studies show the mechanisms by which *T. cruzi* modulates DCs, but the interaction of these cells with the Mexican strains of *T. cruzi* such as Ninoa and INC5 has not yet been properly investigated. Here, we evaluated whether Ninoa and INC5 strains evaded the immunity of their hosts by modulating the biology and function of murine DCs. The CL-Brener strain was used as the reference strain. Herein, it was demonstrated that Ninoa was more infective toward bone marrow-derived dendritic cells (BMDCs) than INC5 and CL-Brener strains in both BMDCs of BALB/c and C57BL/6 mice. Mexican strains of *T. cruzi* induced different cytokine patterns. In BMDCs obtained from BALB/c mice, Ninoa strain led to the reduction in IL-6 and increased IL-10 production, while in C57BL/6 mice Ninoa strain considerably increased the productions of TNF-*α* and IL-10. Also, Ninoa and INC5 differentially modulated BMDC expressions of MHC-II, TLR2, and TLR4 in both BALB/c and C57BL/6 mice compared to Brazilian strain CL-Brener. These results indicate that *T. cruzi* Mexican strains differentially infect and modulate MHC-II, toll-like receptors, and cytokine production in DCs obtained from C57BL/6 and BALB/c mice, suggesting that these strains have developed particular modulatory strategies to disrupt DCs and, consequently, the host immune responses.

## 1. Introduction

Chagas disease, an illness identified 110 years ago by the physician and researcher Carlos Chagas, is a serious public health problem, affecting approximately 8 million people worldwide [1, 2]. *Trypanosoma cruzi*, the etiological agent of the disease, presents intraspecific genetic and phenotypic heterogeneity. Based on phylogenetic, molecular, biochemical, and biological markers, the causative agent of the disease is grouped into six discrete typing units (DTUs) ranging from TcI to TcVI [3–5]. The genetic diversity of *T. cruzi* exerts influence on the biological, clinical, immunological, and epidemiological variation of the disease, and it is also directly related to the establishment of the infection [6, 7]. For example, the TcVI genotype is associated with human Chagas disease in countries of South America, especially north of the equator where several cases of human infection have been reported [4]. Specifically, the CL-Brener strain [8] belongs to the TcVI genotype, and the metacyclic forms derived from this strain are highly invasive *in vitro* and *in vivo* [9–11]. TcI is considered a homogeneous group but contains the largest distribution among the described genotypes and is subdivided into five subgroups (TcIa–TcIe) [5, 12–15]. TcI is the genotype that predominates in Mexico and is responsible for causing most of the clinical manifestations of Chagas disease. The Mexican strain of this genotype has different biological characteristics such as growth, metacyclogenesis, and infectivity *in vitro* and can cause patent and subpatent parasitemia. However, the strains belonging to the same TcI genotype differ in their ability to invade cells and cause infection [16–21]. Experimental studies have shown that although the Mexican strains belong to the same TcI genotype, they present differences in the induction of mortality (0–100%), muscle cell tropism (mainly skeletal and cardiac), and in the inflammatory process generated by the infection. This indicates that biological behavior varies between these strains in the same DTU [16, 17, 19, 20]. Thus, elucidating the underlying mechanisms that generate so many differences even in strains of the same genotype and in the same geographical region is essential for understanding the disease in Mexico.

The *T. cruzi* parasite presents three morphological forms in its biological cycle, and of these, metacyclic trypomastigotes are the infective forms eliminated by triatomines during blood feeding [9, 16, 22–24]. The surface molecules presented by the metacyclic trypomastigotes are fundamental for the interaction of the parasite with the host, and through these surface molecules, the protozoan can be recognized by host defense cells. In this context, dendritic cells (DCs) are one of the preferred targets of the infecting forms of *T. cruzi* [25]. Because of their efficient antigen presentation ability, DCs can detect pathogens and initiate an effective response through a cascade of triggered events that culminates in the presentation of antigen to lymphocytes and activation of a specific and protective immune response [26]. In this process, these cells are activated and direct the host immune response depending on the production of cytokines and the presence and intensity of surface markers that characterize their maturation [27–29]. During antigen presentation, these cells have high expression of molecular markers such as CD80, CD86, and MHC [27, 30–32]. Additionally, cellular migration markers such as CCR7, which are fundamental in the migration process of these cells to the presentation sites, are expressed [27, 33]. Furthermore, various proinflammatory cytokines such as IL-1*β*, IL-12, IL-8, TNF-*α*, and IL-6 are synthesized and assist in the formation of a specific pattern of immune response [27, 34, 35].

The interaction with *T. cruzi* induces inhibition of the expression of important cell activation and cellular maturation markers such as CD80, CD86, MHC, and CD40 [36]. In addition, *T. cruzi* induces a DC death marker called PDL-1 that inhibits the production of proinflammatory cytokines such as IL-12, TNF-*α*, and IL-6 and stimulates the synthesis of anti-inflammatory cytokines such as IL-10 and TGF-*β* targeting a tolerogenic profile where there is less activation of *T. cruzi*-specific T lymphocytes [6, 25, 28, 37, 38]. These immunomodulations by the parasite can vary depending on the *T. cruzi* strain and how they interact with these cells, highlighting the key role of DCs in the development of clinical forms of the disease [6, 7, 37, 39–43]. Although many studies have elucidated the mechanisms by which *T. cruzi* modulates DCs, the interaction of these cells with Mexican strains has not yet been properly investigated. The present study is aimed at investigating the infectivity, expressions of standard recognition receptors and costimulatory molecules, and the production of cytokines in DCs cultured with Mexican strains to understand how they evade the immune responses of their hosts by modulating these cells.

## 2. Material and Methods

### 2.1. Parasites

Two strains of *T. cruzi* from Mexico were used. They were maintained in the laboratory of parasitology of the Federal University of the Triângulo Mineiro, UFTM. Ninoa strain (MHOM/MX/1994) [44] was obtained from xenodiagnosis of a patient with acute Chagas disease, while the INC5 strain (MHOM/MX/1994) [45] was isolated from a patient with Chagasic cardiomyopathy [19, 20]. CL-Brener is a Brazilian strain and was used as a reference for the study. This strain was isolated from a *Triatoma infestans*, belonging to the Department of Parasitology of the Federal University of São Paulo, UNIFESP, and was kindly provided by Dra. Nobuko Yoshida. The parasites were cultured at 28°C in liver infusion tryptose (LIT) medium supplemented with 10% fetal bovine serum. Metacyclic forms of cultures in stationary growth phase were purified by column passing DEAE-cellulose as previously described [46].

### 2.2. Animals and Differentiation of Bone Marrow-Derived DCs (BMDCs)

Male BALB/c and C57BL/6 (6–8 weeks old) wild-type mice were bred and maintained in experimental animal facilities of the Federal University of Triângulo Mineiro, UFTM, Uberaba, MG, Brazil, according to the guidelines of the Ethics Committee on Animal Use (CEUA). All the experiments were conducted according to the Ethics Committee on the Use of Animals of the Federal University of Triângulo Mineiro. Bone marrow cells from the femurs and tibiae removed from mice were centrifuged at 400 × *g* for 10 min at 8°C in RPMI 1640 medium (GE Healthcare, Uppsala, Sweden). Subsequently, 2 mL of lysis buffer was added for lysing the red cells, and the cells were washed thrice for counting. Cells were counted in a Neubauer chamber and resuspended to 5 × 10^6^ cells/mL in RPMI 1640 medium with the addition of 50 mM Hepes (Gibco, Grand Island, NY, USA), 10% of inactivated fetal bovine serum (Gibco, USA), 2 mM L-glutamine (Gibco, USA), 40 mg/mL gentamicin, and 12.5 ng/mL murine GM-CSF (BD) at 37°C in a humidified atmosphere with 5% CO_2_. On day 3, 10 mL of RPMI medium containing 12.5 ng/mL GM-CSF was added. Cells were further differentiated for additional 4 days with GM-CSF containing complete medium. After 7 days of culture, the cells were collected and analyzed by flow cytometry to determine the percentage of CD11b and CD11c, and further experiments were performed only after evaluating this percentage. All cultures presented at least 80% of CD11b^+^CD11c^+^ DCs, and bone marrow-derived dendritic cells (BMDCs) were harvested and cultured in 96-well plates.

### 2.3. *In Vitro* Infection of DCs

After 7 days of differentiation, BMDCs at a concentration of 1.5 × 10^5^ cells per well (96-well plate) in 250 *μ*L of 10% RPMI 1640 medium were incubated for 18 h with the three different *T. cruzi* strains (CL-Brener, Ninoa, or INC5 at MOI 2 : 1) with or without LPS (5 *μ*g/mL). Cells were then evaluated for parasite infection using 4′,6-diamidino-2-phenylindole dihydrochloride (DAPI; Molecular Probes, Eugene, Oregon, US). Expression of MHC-II, CD80, CD86, TLR2, and TLR4 surface markers was evaluated by flow cytometry, and the production of TNF-*α*, IL-10, IL-12p70, CCL-2, and IL-6 was measured by cytometric bead array. A MOI of 2 : 1 was chosen because this ratio of cell and parasite was sufficient to affect the different steps of the DC biology or to assess parasitic infectivity.

### 2.4. Determination of *T. cruzi*-Infected DCs

BMDCs in 96-well plates were analyzed by fluorescence microscopy using DAPI. BMDCs differentiated from both the mouse strains were cultured with different strains of *T. cruzi*, harvested, and incubated at 4°C for 30 min with 10 *μ*M DAPI. Cells were washed twice with PBS and centrifuged at 400 × *g* at 4°C for 10 min and immediately analyzed by EVOS Cell Imaging System (Thermo Scientific, USA) at ×400 magnification. The number of intracellular parasites was counted in a total of 100 cells, and percentage of infected cells, parasites per 100 cells, and mean parasite load in infected cells were determined.

### 2.5. Flow Cytometry Analysis

BMDCs differentiated from both mouse strains were analyzed by flow cytometry using the following monoclonal antibodies: anti-CD11c, anti-MHC-II, anti-CD80, anti-CD86, anti-TLR2, or anti-TLR4, labeled with APC, FITC, PE, or PECy7 according to the intended purpose. The acquisition was obtained using an Accuri flow cytometer (BD Immunocytometry Systems), and the analyses were performed using the FlowJo software.

### 2.6. Cytometric Bead Array

Cytometric bead array was performed using the CBA Mouse Inflammation Kit (BD Biosciences) according to the manufacturer's instructions, and the following cytokines were measured: TNF-*α*, IL-10, IL-12p70, IL-6, and chemokine CCL-2. Briefly, supernatants of BMDCs from C57BL/6 and BALB/c mice cultured with *T. cruzi*, with or without LPS, were incubated with beads coupled with specific monoclonal antibodies and PE-conjugated secondary antibodies for 4 h at room temperature. Beads were washed and the acquisition was performed using the Accuri flow cytometer (BD Immunocytometry Systems). The concentration of the samples was estimated by comparing the PE fluorescence obtained from the standard curve obtained by serial dilution of recombinant murine cytokines. Results were analyzed by 5-parameter logistic regression with FCAP array software and expressed in pg/mL.

### 2.7. Statistical Analysis

The results were analyzed by GraphPad Prism 7.0 (GraphPad Software, San Diego, CA, USA). The Kruskal-Wallis test with a Dunn's post hoc test was performed for data with a non-Gaussian distribution. Bar graphs show mean and standard error of the mean. The results were considered significant when the *p* value was lower than 0.05 (5%).

## 3. Results

### 3.1. Evaluation of the Infectivity of Different Strains of *T. cruzi* in DCs Derived from BALB/c and C57BL/6 Mice

**Figure 1:**
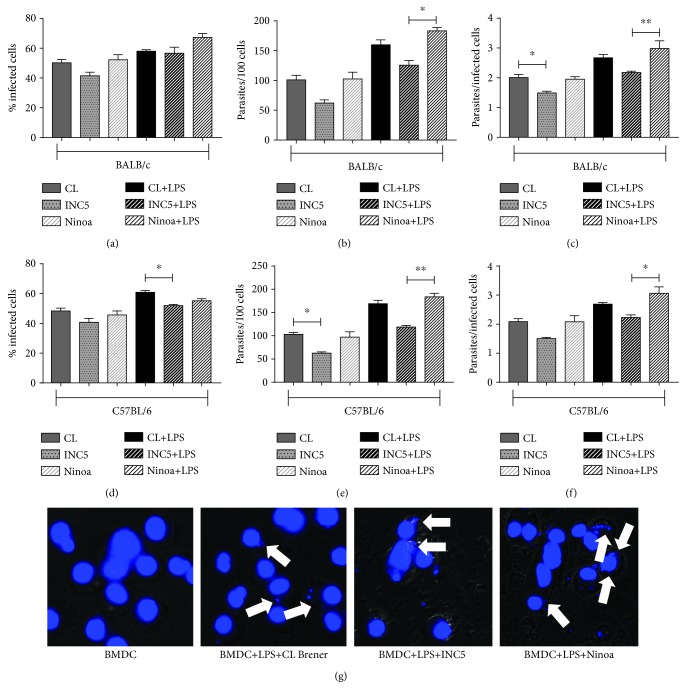
*Trypanosoma cruzi* infectivity in murine BMDCs from BALB/c and C57BL/6 mice. BMDCs with or without LPS stimulation (5 *μ*g/mL) were incubated for 18 h with different *T. cruzi* strains (MOI 2 : 1) and stained with DAPI. (a) and (d) show the percentage of infected cells; in (b) and (e) the number of parasites per 100 cells and in (c) and (f) the ratio of parasites per infected cell are shown. (g) Representative images of BMDCs infected with *T. cruzi* after stimulation with LPS (5 *μ*g/mL) and stained with DAPI. Statistical analysis, when applicable, was performed with the Kruskal-Wallis test with Dunn's posttest, where ^∗,∗∗^*p* < 0.05.

In general, the different *T. cruzi* strains were able to infect DCs. However, the percentage of infection varied depending on the strain and the origin of DCs [6, 7, 37, 47]. In BMDCs derived from BALB/c mice, the percentage of cells infected with the different strains did not show a significant difference despite experiencing a slight increase when these cells were cultured with LPS (Figure 1(a)). Contrastingly, when evaluating the amount of parasites present in a total of 100 BMDCs, we found that cells cultured with *T. cruzi* from the Ninoa strain previously stimulated with LPS exhibited a significant increase in the number of parasites compared to the INC5 strain plus LPS (*p* = 0.0427) (Figure 1(b)). The same profile in relation to the Ninoa and INC5 strains plus LPS was found by analyzing the amount of parasites present in each infected cell (*p* = 0.0219) (Figure 1(c)). In the CL-Brener strain without LPS treatment, the number of parasites per infected cell was significantly higher compared to the INC5 strain also without LPS treatment (*p* = 0.0427) (Figure 1(c)). When the BMDCs derived from C57BL/6 mice were evaluated, the percentage of infected cells after culture with CL-Brener strain plus LPS presented a significant variation (*p* = 0.0234) compared to BMDCs cultured with INC5 strain plus LPS (Figure 1(d)). The amount of parasites per 100 cells showed a significant increase when the CL-Brener strain was compared to the INC5 strain (*p* = 0.0422). The same difference in infectivity was found when the number of parasites in BMDCs cultured with the CL-Brener strain plus LPS was compared to BMDCs cultured with the Ninoa strain plus LPS (*p* = 0.0230) (Figure 1(e)). The same pattern was observed in the amount of parasites per infected cell comparing the effects of the Ninoa and CL-Brener strains (*p* = 0.0181) (Figure 1(f)). In Figure 1(g), we show a representative picture of DAPI labeling evidencing the infectivity of different strains of *T. cruzi* in BMDCs *in vitro*.

### 3.2. Production of Cytokines and Chemokine CCL-2 in DCs Cultured with Different Strains of *T. cruzi*

**Figure 2:**
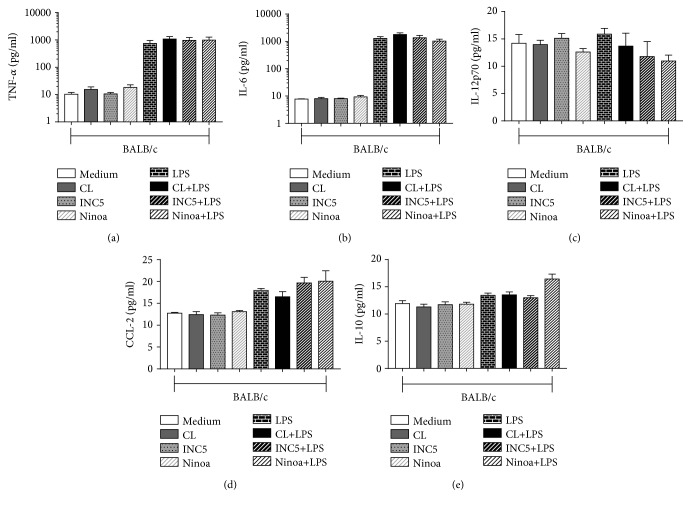
Cytokine and chemokine production by BALB/c-derived BMDCs infected with CL-Brener, Ninoa, and INC5 strains of *T cruzi*. BMDCs with or without LPS stimulation (5 *μ*g/mL) were incubated for 18 h with different *T. cruzi* strains (MOI 2 : 1), and the production of cytokines and chemokine was evaluated by CBA: (a) TNF-*α*, (b) IL-6, (c) IL-12p70, (d) CCL-2, and (e) IL-10. Statistical analysis, when applicable, was performed with the Kruskal-Wallis test with Dunn's posttest, where ^∗,∗∗^*p* < 0.05.

**Figure 3:**
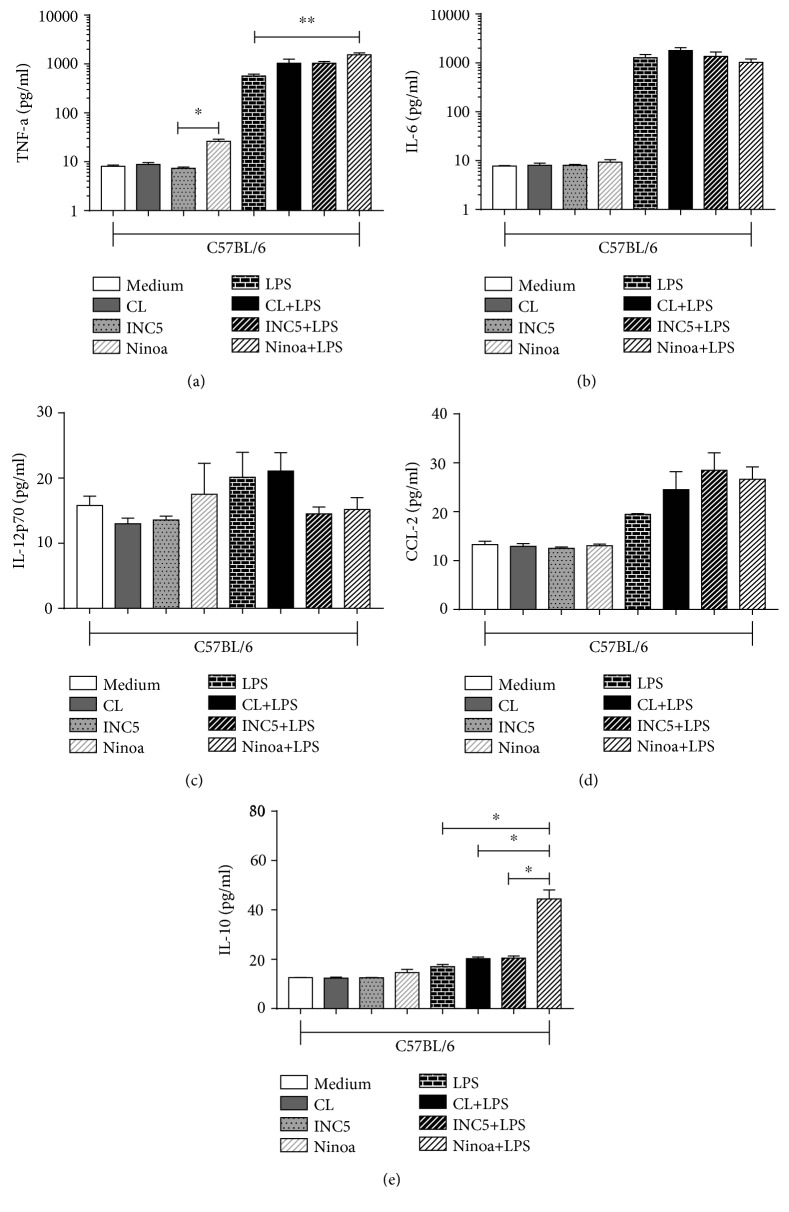
Cytokine and chemokine production by C57BL/6-derived BMDC infected with CL-Brener, Ninoa, and INC5 strains of *T cruzi*. BMDCs with or without LPS stimulation (5 *μ*g/mL) were incubated for 18 h with different *T. cruzi* strains (MOI 2 : 1), and the production of cytokines and chemokine was evaluated by CBA: (a) TNF-*α*, (b) IL-6, (c) IL-12p70, (d) CCL-2, and (e) IL-10. Statistical analysis, when applicable, was performed with the Kruskal-Wallis test with Dunn's posttest, where ^∗,∗∗^*p* < 0.05.

The concentration of cytokines in BMDCs from BALB/c mice did not show statistically significant differences in any of the analyses, regardless of whether they were infected only with the strains or in the presence of LPS. The following cytokine levels were measured: TNF-*α*, IL-6, IL-12p70, CCL-2, and IL-10 (Figures 2(a)–2(e)). When we analyzed the cytokines present in the culture supernatant of BMDCs derived from C57BL/6 mice, a significant increase was observed in the production of TNF-*α* in BMDCs cultured with the Ninoa strain without or plus LPS compared to cultures without *T. cruzi* infection (*p* = 0.0094) (Figure 3(a)). Also, the Ninoa strain induced a significant increment of IL-10 production in LPS-treated cultures compared to LPS alone and all other strains plus LPS (*p* = 0.00001 for LPS, *p* = 0.023 for CL-Brener, and *p* = 0.0331 for INC5) (Figure 3(e)). We did not find significant differences in the production of IL-6 (Figure 3(b)), IL-12p70 (Figure 3(c)), and CCL-2 (Figure 3(d)).

### 3.3. Percentage of Cytokine and Chemokine CCL-2 Variation in DCs Cultured with Different Strains of *T. cruzi*

**Figure 4:**
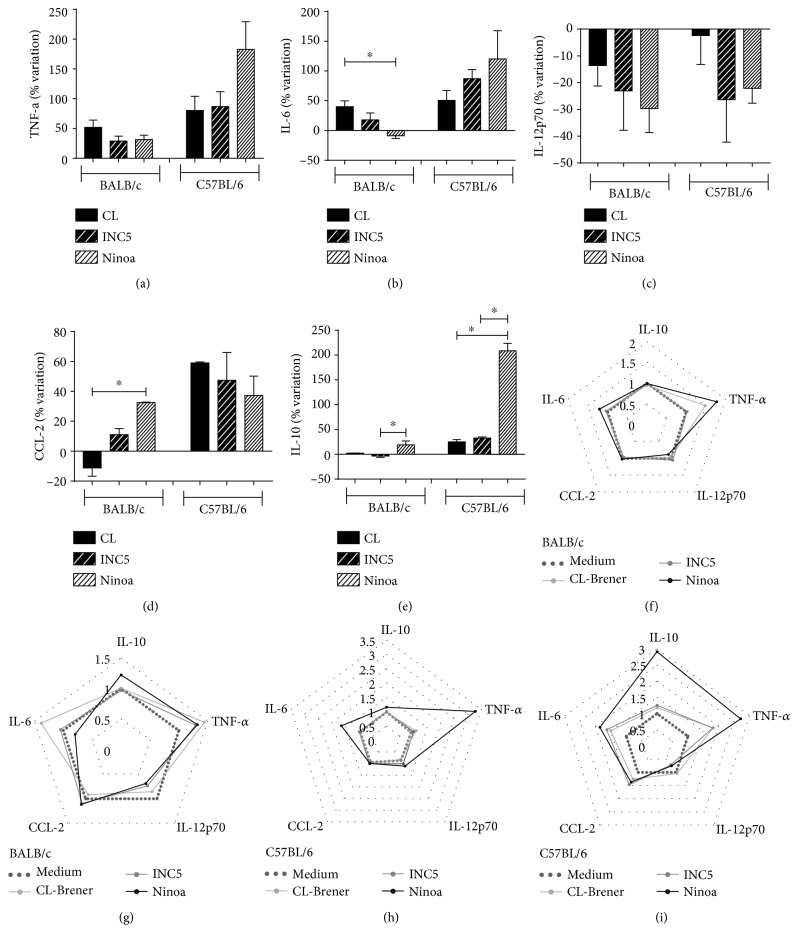
Percentage of variation in the levels of pro-and anti-inflammatory cytokines and CCL-2 chemokine in the supernatant of BMDCs of BALB/c and C57BL/6 mice. The variation of cytokines was calculated by determination of percentage of increment or reduction in cytokines produced by *T. cruzi-*infected BMDCs previously stimulated with LPS and those maintained only with LPS (5 *μ*g/mL). (f, h) Representation of the radar graph of the cytokine pattern in the dendritic cell culture supernatant. The lines highlight the change in cytokine levels in dendritic cells infected with different strains (MOI 2 : 1) in relation to the medium only with dendritic cells. (g, i) The pattern of cytokines in the dendritic cell culture supernatant previously stimulated with LPS. Data were obtained by calculating the ratio between *T. cruzi*-infected cells and their respective control. Statistical analysis, when applicable, was performed with the Kruskal-Wallis test with Dunn's posttest, where ^∗,∗∗^*p* < 0.05.

In order to demonstrate how the different *T.* cruzi strains could modulate the ability of dendritic cells to produce cytokines, we calculated the variation of cytokines by determination of percentage of increment or reduction in cytokines produced by *T. cruzi*-infected BMDCs previously treated with LPS and those maintained only with LPS (5 *μ*g/mL). Globally, the interaction of BMDCs with *T. cruzi* induced TNF-*α*, IL-6, CCL-2, and IL-10 increment and IL-12p70 reduction. The cytokine IL-6 (Figure 4(b)) showed significant alteration only in the BMDCs derived from BALB/c mice, showing a reduction in cells stimulated with the Ninoa strain compared to cells stimulated with the CL-Brener strain (*p* = 0.0387). On the other hand, the Ninoa strain induced significant increment in CCL-2 compared to CL-Brener in BALB/c mice (*p* = 0.01). IL-10 also showed significant variation for both mice strains, as BMDCs cultured with the Ninoa strain had an increase in IL-10 compared to BMDCs cultured with the INC5 strain in BALB/c mice (*p* = 0.0219) and compared to INC5 and CL-Brener in C57BL/6 mice (*p* < 0.0001). We did not observe any significant changes in the variation of TNF-*α* (Figure 4(a)) and IL-12p70 among strains (Figure 4(c)). In Figures 4(f)–4(i), it is possible to observe the variation in the production of the cytokines according to the different strains with or without the addition of LPS.

### 3.4. Evaluation of MHC-II, CD80, CD86, TLR2, and TLR4 Expression in DCs Cultured with Different Strains of *T. cruzi*

**Figure 5:**
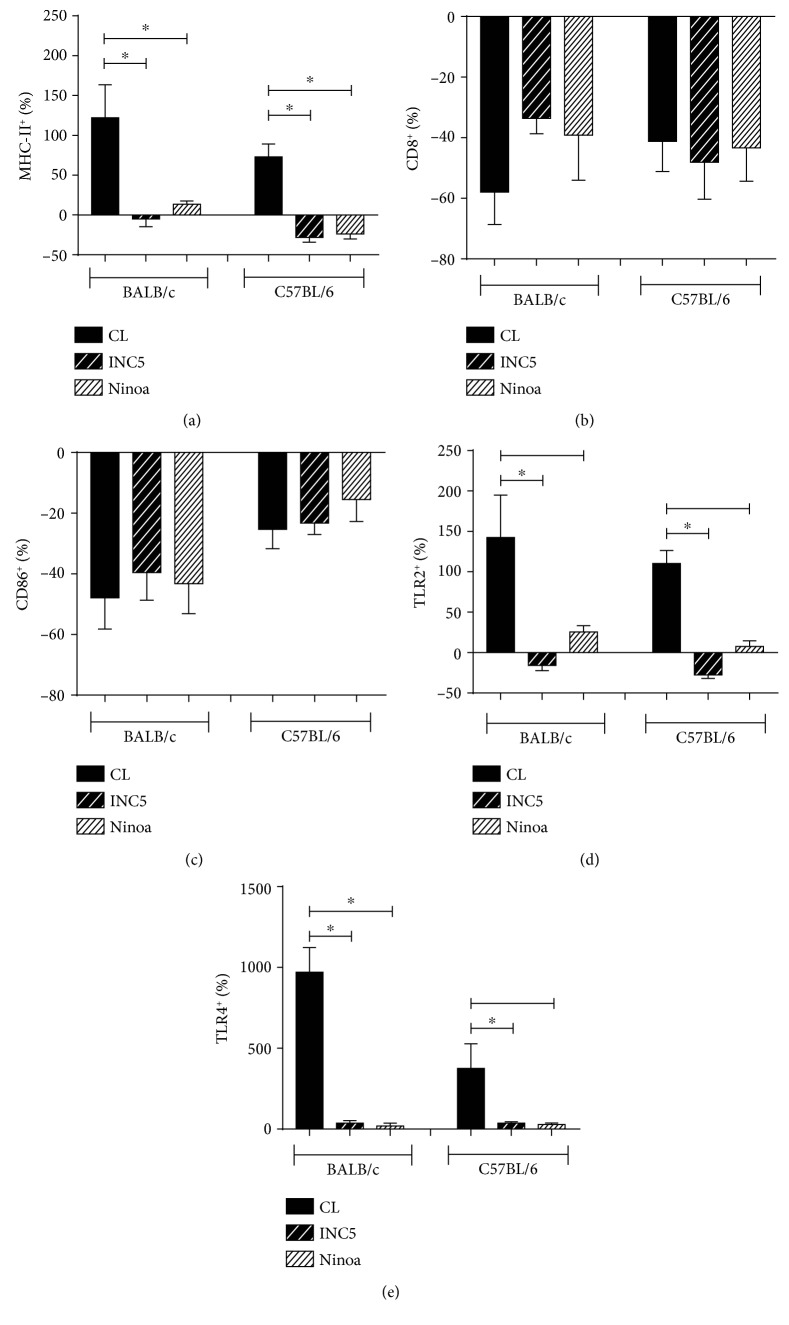
Percentage of BMDCs expressing MHC-II, costimulatory molecules, and toll-like receptors after *in vitro T. cruzi* infection. The expression of molecules was evaluated by flow cytometry and represented as a variation of the percentage of BMDCs expressing (a) MHC-II, (b) CD80, (c) CD86, (d) TLR2, and (e) TLR4. Statistical analysis, when applicable, was performed with the Kruskal-Wallis test with Dunn's posttest, where ^∗^*p* < 0.05.

Data related to flow cytometry were analyzed by ascertaining the variation of the percentage of positive cells and the variation of the expression per cell (mean fluorescence intensity). Our results point that *T. cruzi* Mexican strains significantly reduce cells expressing MHC-II, TLR2, and TLR4 compared to the Brazilian strain CL-Brener. Specifically, the interaction with INC-5 and Ninoa led to a lower MHC-II^+^ BMDC in both BALB/c (*p* = 0.0011) and C57BL/6 (*p* = 0.0004) mice (Figure 5(a)). The costimulatory molecules CD80 and CD86 presented a negative variation in BMDCs cultured with all evaluated (Figures 5(b) and 5(c)). For both BALB/c and C57BL/6, the CL-Brener strain induced higher percentage of TLR2^+^ BMDCs compared to the INC5 strain (*p* = 0.0051 and *p* = 0.0001, respectively) and to Ninoa (*p* = 0.0051 and *p* = 0.0001, respectively, Figure 5(d)). In a similar way, the CL-Brener strain interaction induced a higher percentage of TLR4^+^ BMDCs in both mice strains compared to the INC5 (*p* = 0.0001 for BALB/c and C57BL/6) and Ninoa strains (*p* = 0.0001 for BALB/c and C57BL/6) (Figure 5(e)).

**Figure 6:**
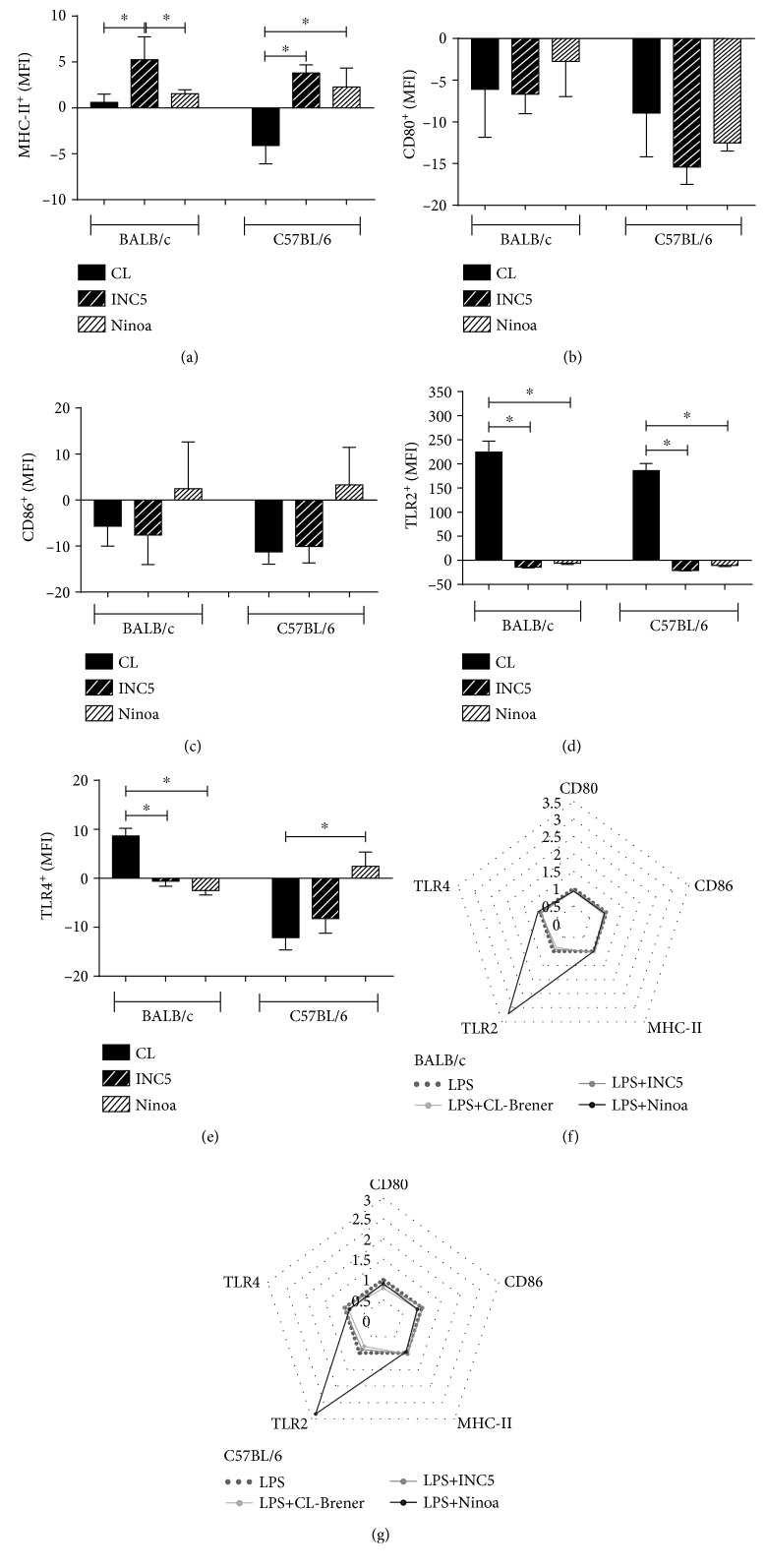
Expression of MHC-II, costimulatory molecules, and toll-like receptors in BMDCs after *in vitro T. cruzi* infection. The expression of molecules was evaluated by flow cytometry and represented as a variation of the mean intensity of fluorescence (a) MHC-II, (b) CD80, (c) CD86, (d) TLR2, and (e) TLR4. (f, g) Representation of the cytokine secretion pattern in BMDC culture supernatant. The lines highlight the change in cytokine levels in LPS-stimulated BMDCs and infected with different strains of *T. cruzi* (MOI 2 : 1) in relation to uninfected LPS-stimulated BMDCs. Statistical analysis, when applicable, was performed with the Kruskal-Wallis test with Dunn's posttest, where ^∗^*p* < 0.05.

In both mouse strains, the INC5 strain led to a significant increment in BMDC MHC-II expression compared to the CL-Brener strain (*p* = 0.0319) and Ninoa (*p* = 0.0004) in BALB/c. Interestingly, while in BALB/c mice the CL-Brener strain induced a slight increment in MHC-II, in C57BL/6 mice, this expression was inhibited and significantly different from INC5 and Ninoa (*p* = 0.0427, Figure 6(a)). All *T. cruzi* strains induced an inhibition in CD80 expression for both mouse strains (Figure 6(b)). For CD86, only Ninoa induced a slight increment in expression but without statistical significance (Figure 6(c)). The CL-Brener strain also induced a considerable increment in TLR2 expression in both mouse strains compared to the INC5 and Ninoa strains (*p* = 0.0002 for BALB/c and *p* = 0.0001 for C57BL/6, Figure 6(d)). On the other hand, the CL-Brener strain infection induced a lower expression of TLR4^+^ BMDCs compared to the INC5 and Ninoa strains (*p* = 0.0004 for BALB/c and *p* = 0.01 for C57BL/6, Figure 6(d)). Radar plots present a summary of the impact of the interaction with different *T. cruzi* strains in LPS-stimulated BMDCs from BALB/c (Figure 6(f)) and C57BL/6 (Figure 6(g)) mice.

## 4. Discussion

Our study is aimed at evaluating the infectivity and immunomodulatory capacity of different Mexican strains of *T. cruzi* on DCs derived from BALB/c and C57BL/6 mice. Our results point to the fundamental role of these strains in the interaction with these DCs during the *in vitro* coculture, depending on the mouse lineage.

The biological behavior, anatomical route of invasion, inoculum, surface molecules expressed in the metacyclic forms of *T. cruzi*, and host immune response are factors that are closely related to the establishment of the infection [6, 9, 48–50]. Our data showed that all three strains infected DCs efficiently. However, strains belonging to the TcI genotype (AQ1.7, Mutum, and G) presented a profile with low infectivity in BMDCs [6, 51]. This can be explained by the fact that *T. cruzi* presents a high intraspecific genetic and phenotypic diversity, especially for the TcI genotype. Thus, different genetic markers indicate that there is an intra-DTU genetic variation, and the TcI is the genotype that presents high genetic heterogeneity. This may be related to the different epidemiological characteristics and generate controversies regarding the infectivity and pathogenicity of the strains belonging to this genetic group [4, 13, 19, 52].

When inoculated in Swiss mice, the parasites of the INC5 and Ninoa strains presented patent parasitemia, high infectivity, and mortality. Intense tissue parasitism was observed in several organs of the experimental animals, increasing the virulence of these strains [20, 52]. However, trypomastigote forms were used in these other studies [17, 20, 21]. Notably, the different evolutionary forms of *T. cruzi* use different strategies in adhesion or invasion of cells because of the specific stage molecules, which affect the infectivity and pathogenicity, even in the same strain [53, 54]. The parasite inoculation pathway can also influence the biological behavior of the strain because of the different biological barriers that the parasite needs to overcome to establish the infection, directly impacting the immune response and host resistance [50]. One factor that needs to be considered is the type of cell or tissue that the metacyclic form infects. The Ninoa strain showed low parasitemia and mortality and a mild inflammatory process when inoculated orally in mice of this same strain. In oral infection, the parasites enter into the epithelial cells of the stomach and can undergo pepsin action, and the metacyclic trypomastigote forms express on their surface molecules that can facilitate or inhibit this invasion process. Recent studies have shown that the TcI genotype has a poor infective oral profile, as they express gp90 on their surface, and this molecule negatively regulates invasion to the target cell [16, 55].

On the other hand and consistent with our present study, the CL-Brener strain presents a highly infectious profile in *in vitro* studies, and its profile is well studied in oral infection. An explanation for the increased infectivity may be the glycoprotein gp82 expressed on its surface that facilitates the entry of the parasite into the cell through the mobilization of intracellular calcium [9, 11]. These different results using the same strains show that in addition to the parasite genetics, the infected cell line and the immune response caused by the contact of the parasite with the cell are associated with the infection. Additionally, the cells derived from the C57B/6 and BALB/c mice present different degrees of susceptibility and/or resistance [51]. Our results show that the Ninoa strain has a higher infectivity potential when the BMDCs are activated with LPS, while the CL-Brener strain is more infective in BMDCs not yet activated. Different strains have shown distinct infectivity potentials [6], and this can be related to the ability of these cells to recognize the molecules presented on the surface of the parasite [56]. In addition, in BALB/c mice, we observed a similar cell interaction for all strains, while in C57BL/6 the CL-Brener strain had a better performance. This suggests that a strain can have different behaviors depending on the mice used, as shown in experiments with Y strain [57].

Once the ability of infection in cells derived from the two strains was confirmed, we analyzed the ability of these strains to modulate surface markers and stimulate the production of cytokines. We observed that both strains of *T. cruzi* and lineage of mice presented different patterns for the parameters evaluated. In BMDC from BALB/c mice, the Ninoa strain lead to a reduction in IL-6 and an increase in IL-10 production, while in C57BL/6 mice the cytokine production was associated with a huge increment in TNF-*α* and IL-10. Cytokines are one of the most relevant factors in determining the course of infection [17, 51, 58]. During the process of infection by *T. cruzi*, a modulating response associated with susceptibility to the disease can be triggered by the parasite. For instance, in macrophages infected with *T. cruzi*, the activation of TLR is weak, leading to a decrease in the production of proinflammatory cytokines such as IL-12 [59]. In addition, the production of IL-10 and TGF-*β* is stimulated in infected macrophages, and this causes a favorable response to the parasite to be triggered [37, 60, 61]. Similar results were found in our study, where the Ninoa strain presented more modulatory behaviors compared to the other strains under similar conditions, especially when the cells were already activated. According to Gil-Jaramillo et al. [7], more virulent strains can manipulate DCs to produce more tolerogenic cytokines.

Ferreira et al. [51] described increased levels of IL-10 in C57B1/6 and BALB/c mice in the acute phase of the disease. In addition, BALB/c mice had elevated serum TGF-*β* levels, indicating susceptibility to the disease. The proinflammatory cytokines TNF-*α*, IFN-*γ*, IL-1*β*, IL-2, IL-5, and IL-6 also showed high levels of expression during the infection period, suggesting that the balance between cytokines determines the course of infection. In DCs derived from BALB/c mice, we observed that no cytokine showed great peak expression; however, in C57BL/6, IL-10 and TNF-*α* were strongly stimulated by the Ninoa strain. The other cytokine levels did not change. Analyzing the serum of BALB/c animals infected with the Ninoa strain, the study conducted by Espinoza et al. demonstrated an increase in the levels of IL-10 in the acute phase, which decays and rises gradually during the course of infection. IL-12, which reached its peak in the acute phase and was maintained throughout the chronic phase, was also evaluated [17].

TLRs form a family of transmembrane proteins responsible for the recognition of molecular patterns essential for the triggering of immune responses [62–64]. Several surface molecules of *T. cruzi* are recognized by these receptors. Some molecules of trypomastigotes are recognized by TLR2 [65, 66], and other molecules present in epimastigotes are recognized by TLR4 [67]. The relationship between the recognition of *T. cruzi* by these receptors and the development of a susceptible or resistant immune response has been extensively investigated. TLR2, TLR4, TLR7, and TLR9 are important for the development mechanisms of *T. cruzi* infection [64]. The parasitic form tested in our study for all the strains used was the metacyclic, the same as that of the natural infections in vector transmission, and we observed a better expression of TLR2 in cells infected by the CL-Brener strain belonging to TcVI in relation to the INC5 strain belonging to TcI and a better expression of TLR4 in relation to the Ninoa strain in the TcI group. Similar studies using other strains showed strains belonging to the TcI group that were able to improve TLR2 expression, whereas the decrease in TLR4^+^ was evidenced in the TcII group [6]. The increase in TLR2 can be related to the ability to evade host defense by changing the cytokine profile produced, favoring the production of TLR2-dependent IL-10, as in infections by several other microorganisms [6, 51, 59, 67, 68]. Although this strain has not been highlighted by the production of IL-10 compared to others, the TLR2 pathway may be responsible for the observed production. TLR4 can trigger the production of cytokines such as IL-12, IFN-*γ*, TNF-*α*, and nitric oxide (NO) in *T. cruzi* infections [67, 69], leading to better response. Our data show that the CL-Brener strain presents a greater number of TLR4^+^ DCs, although not showing a significant expression with respect to the cytokines of these profiles.

The ability of maturation and presentation of antigens by DCs is impaired by the presence of the parasite. This damage is due to the modulation of important molecules such as MHC, CD80, CD40, and CD86, whose levels are reduced in the presence of the parasite [6, 25, 28, 37, 38]. Only the INC5 strain induced high amounts of MHC-II. In general, this molecule is responsible for the success of antigen presentation and efficient assembly of specific immune response, including the production of antibodies [27, 30–32]. This is consistent with the data shown by Henrique et al., indicating that the most virulent strains Ninoa, INC5, and Colombian induced high production of antibodies. The costimulatory molecules CD80 and CD86 are also related to the maturation and presentation capacity of the antigen [27, 30–32, 52]. The strains studied here presented a negative variation for CD80 and CD86 in BMDCs derived from the two mouse lineages, suggesting a difficulty of maturation of these infected cells. A similar result was observed with splenic DCs infected with the Tehuantepec strain, where the decrease in CD86 expression prevented the migration of these cells to the antigen-presenting organs [47]. This represents a good escape strategy for the host defense parasite.

The metacyclic form is the infective form of transmission of *T. cruzi*, and the first cells found after infection are DCs. However, many studies on these cells were not performed using this infective form [6]. Thus, the evaluation of our dataset allows us to observe situations that can mimic what occurs during the interaction process with the host. By using strains of *T. cruzi* and different mice, we were able to expand the observations even further and conclude that the two Mexican strains studied here were capable of modulating the response of DCs, regardless of their origin, through different pathways. The results reinforce the escape strategy of the host immune response and demonstrate the importance of investigating the mechanisms involved in this process.

## Data Availability

The datasets generated during and/or analyzed during the current study are available from the corresponding author on reasonable request.
